# Laparoscopic *versus* open surgery for rectal cancer: individual patient data meta-analysis of the ALaCaRT and Z6051 randomized trials

**DOI:** 10.1093/bjsopen/zrag119

**Published:** 2026-08-07

**Authors:** Kilian G M Brown, Andrew R L Stevenson, Michael J Solomon, James Murray, Kate Wilson, Val Gebski, James Fleshman, Qian Shi, Thomas E Read, John Simes

**Affiliations:** Department of Colorectal Surgery, Royal Prince Alfred Hospital, Sydney, New South Wales, Australia; Surgical Outcomes Research Centre (SOuRCe) and Institute of Academic Surgery, Royal Prince Alfred Hospital, Sydney, New South Wales, Australia; Faculty of Medicine and Health, Central Clinical School, University of Sydney, Sydney, New South Wales, Australia; Department of Colorectal Surgery, Royal Brisbane and Women’s Hospital, Brisbane, Queensland, Australia; Faculty of Health, Medicine and Behavioural Sciences, University of Queensland, Brisbane, Queensland, Australia; Faculty of Medicine and Health, National Health and Medical Research Council Clinical Trials Centre, University of Sydney, Camperdown, New South Wales, Australia; St Vincent’s Private Hospital Northside, Brisbane, Queensland, Australia; Department of Colorectal Surgery, Royal Prince Alfred Hospital, Sydney, New South Wales, Australia; Surgical Outcomes Research Centre (SOuRCe) and Institute of Academic Surgery, Royal Prince Alfred Hospital, Sydney, New South Wales, Australia; Faculty of Medicine and Health, Central Clinical School, University of Sydney, Sydney, New South Wales, Australia; Faculty of Medicine and Health, National Health and Medical Research Council Clinical Trials Centre, University of Sydney, Camperdown, New South Wales, Australia; Surgical Outcomes Research Centre (SOuRCe) and Institute of Academic Surgery, Royal Prince Alfred Hospital, Sydney, New South Wales, Australia; Faculty of Medicine and Health, Central Clinical School, University of Sydney, Sydney, New South Wales, Australia; Faculty of Medicine and Health, National Health and Medical Research Council Clinical Trials Centre, University of Sydney, Camperdown, New South Wales, Australia; Faculty of Medicine and Health, National Health and Medical Research Council Clinical Trials Centre, University of Sydney, Camperdown, New South Wales, Australia; Department of Surgery, Baylor University Medical Center, Dallas, Texas, USA; Division of Clinical Trials and Biostatistics, Mayo Clinic, Rochester, Minnesota, USA; Department of Surgery, University of Hawaii John A. Burns School of Medicine, Honolulu, Hawaii, USA; Faculty of Medicine and Health, National Health and Medical Research Council Clinical Trials Centre, University of Sydney, Camperdown, New South Wales, Australia

**Keywords:** total mesorectal excision, laparoscopic surgery, proctectomy

## Abstract

**Background:**

The multicentre ALaCaRT and ACOSOG Z6051 randomized trials were unable to demonstrate non-inferiority of laparoscopic *versus* open surgery for rectal cancer with respect to a composite pathology metric indicating successful resection. Neither trial was individually powered to detect differences in long-term recurrence or survival. This planned meta-analysis determined long-term oncological outcomes of laparoscopic *versus* open proctectomy for rectal adenocarcinoma.

**Methods:**

This prospective meta-analysis included individual patient data from patients with cT1–3 N0–2 M0 rectal adenocarcinoma enrolled in the ALaCaRT and ACOSOG Z6051 trials. Pathologically successful resection was defined as complete or near-complete total mesorectal excision, a clear circumferential resection margin (CRM; > 1 mm), and a clear distal resection margin (> 1 mm). The non-inferiority margin for disease-free survival (DFS) was an absolute difference of 5% at least 3 years after surgery.

**Results:**

The combined data set included 935 patients (65.6% men, mean age 60.7 years, mean body mass index 26.7 kg/m^2^) randomized to open (457 patients) or laparoscopic (478 patients) proctectomy. Pathologically successful resection was lower in the laparoscopic than open group (85.1% *versus* 89.9%, respectively; pooled estimate 4.6% difference; 95% confidence interval (c.i.) −8.6% to −0.5%). The median follow-up was 60.2 (interquartile range 49.9–61.1) months. Three-year DFS was 75.2% (95% c.i. 71.1% to 79.2%) and 76.5% (95% c.i. 72.5% to 80.6%) for the laparoscopic and open groups, respectively (pooled estimate difference −1.5%; 95% c.i. −7.2% to 4.2%). Non-inferiority of laparoscopic surgery was not demonstrated because the lower one-sided 95% c.i. (−6.3% to 100%) crossed −5%. Three-year locoregional recurrence was higher in laparoscopic than open group (5.4% (95% c.i. 3.3% to 7.5%) *versus* 2.0% (95% c.i. 0.7% to 3.4%), respectively; pooled estimate 3.1% difference (95% c.i. 0.6% to 5.6%)). A clear CRM was the most significant and only pathological predictor of both DFS (*P* < 0.0001) and overall survival (*P* < 0.0001).

**Conclusion:**

Laparoscopic proctectomy led to a lower rate of pathologically successful resection and a higher rate of locoregional recurrence at 3 years. The possibility of subsequent poorer DFS or overall survival rate requires further evaluation, because this analysis was not specifically powered for these endpoints.

## Teaser

This individual patient data meta-analysis of the ALaCaRT and Z6051 randomized trials with 935 patients demonstrates that laparoscopic proctectomy is associated with a lower rate of the combined pathology metric indicating a successful resection, but a higher rate of locoregional recurrence at 3 years. A subsequent poorer disease-free or overall survival rate is not excluded.

## Introduction

The oncological outcomes of surgery for rectal adenocarcinoma are directly influenced by the technical precision of proctectomy using total mesorectal excision (TME). Achieving clear resection margins and an adequate lymphovascular clearance with an intact mesorectal fascia (‘complete TME’) is associated with improved rates of locoregional recurrence (LRR)^1–3^. Whether an equivalent combined pathology metric indicating successful resection and therefore long-term oncological outcomes can be achieved by laparoscopic proctectomy for rectal cancer compared with open pelvic dissection remains unclear.

The Australasian Laparoscopic Cancer of the Rectum Trial (ALaCaRT) and the American College of Surgeons Oncology Group (ACOSOG) Z6051 trial compared the efficacy of open *versus* laparoscopic mesorectal mobilization during proctectomy for rectal adenocarcinoma. Both trials used a composite primary endpoint defined by completeness of mesorectal excision and clear resection margins (circumferential and distal), and both were unable to demonstrate non-inferiority of the laparoscopic approach with respect to this short-term surrogate marker of oncological outcome^4,5^. Although 2-year results from both trials demonstrated similar rates of LRR and disease-free survival (DFS) among laparoscopic and open groups^6,7^, neither trial was powered to detect a difference in these secondary endpoints. Therefore, these findings do not exclude the possibility of inferior oncological outcomes after laparoscopic proctectomy, particularly given that the estimate of treatment effect favoured open resection in both trials.

The ALaCaRT and Z6051 trials were designed simultaneously with consultation between trial committees, using similar inclusion criteria and outcome measurements. A prospective meta-analysis of individual patient data (IPD) was planned at the time of design of both trials and was to be conducted after recruitment and follow-up were complete for both trials, where a larger sample (expected 950 patients) would allow assessment of disease recurrence and survival to be evaluated at a minimum 3-year follow up. This study reports the results of that planned meta-analysis using IPD, including the long-term oncological outcomes (3-year LLR, 3-year DFS, and 5-year overall survival (OS)) of laparoscopic *versus* open proctectomy for rectal adenocarcinoma, with an additional aim of evaluating the validity of the chosen short-term pathological outcomes as surrogate markers of these longer-term oncological outcomes.

## Methods

### Study design

This is a prospective meta-analysis of individual patient data from patients with primary rectal adenocarcinoma enrolled in the multicentre, randomized, controlled ALaCaRT^4,7^ and ACOSOG Z6051^5,6^ trials. These trials were designed by independent trial committees but with cross-consultation and planned similarities defined *a priori*. The participant eligibility criteria, interventions, and planned analyses for the individual trials have been reported previously and are included in the *Supplementary material*. Both trials were conducted in accordance with institutional ethical standards and the Declaration of Helsinki of 1975. Ethics approval and informed consent were obtained as reported previously (Z6051: institutional review board-approved consent; ALaCaRT: Sydney Local Health District Human Research Ethics Committee)^4–7^. Briefly, ALaCaRT (ACTRN12609000663257) was a phase III randomized non-inferiority trial that compared laparoscopic-assisted *versus* open surgery for rectal cancer and was conducted across 24 centres in Australia and New Zealand. In all, 475 patients with adenocarcinoma of the rectum (stage T1–3 N0–2 M0–M1) within 15 cm of the anal verge were randomly assigned to laparoscopic or open proctectomy with TME dissection. Z6051 (NCT00726622) was a phase III randomized non-inferiority trial that compared laparoscopic-assisted *versus* open surgery for rectal cancer and was conducted across 35 centres in the USA and Canada. In all, 486 patients with adenocarcinoma of the rectum (stage T1–3 N0–2 M0) within 12 cm of the anal verge were randomly assigned to laparoscopic or open proctectomy with TME dissection. All patients in Z6051 underwent neoadjuvant long-course chemoradiotherapy and were recommended adjuvant chemotherapy selectively, whereas in ALaCaRT the use of (neo)adjuvant radiotherapy and chemotherapy were recommended by treating clinicians according to local practice, including consideration of patient preferences.

Strict eligibility criteria for treating surgeons were fulfilled in order to be credentialed for both trials. This involved submitting evidence of a minimum number of previous laparoscopic colonic resections (100 for ALaCaRT, 20 for Z6051) and previous laparoscopic rectal resections (30 for ALaCaRT, 20 for Z6051). Surgeons were required to submit operative and pathology reports for all rectal dissections used for credentialing, as well as an unedited video of their laparoscopic proctectomy technique, which was reviewed independently by designated investigators. All laparoscopic procedures conducted in both trials were recorded and randomly audited.

For both trials, follow-up assessments were conducted at 3, 6, 9, 12, 18, and 24 months after surgery and involved physical examination and carcinoembryonic antigen at each visit, as well as yearly computed tomography of the abdomen, pelvis and chest (or plain chest radiograph in ALaCaRT). Follow-up beyond 24 months was yearly in ALaCaRT until 5 years and every 6 months in Z6051 for as long as possible. Where these tests were equivocal, patients with possible recurrence were further evaluated by positron emission tomography or biopsy for histological confirmation.

### Outcomes

The primary outcome of this prospective meta-analysis was disease-free survival (DFS) at a minimum 3-year follow up. Other common endpoints used in ALaCaRT and Z6051 were the rate of pathologically successful resection, LRR, and overall survival (OS), and these are reported as secondary endpoints. Both individual trials used a composite pathology endpoint of pathologically ‘successful’ resection that incorporated completeness of TME, circumferential resection margin (CRM) status, and clear distal resection margin (DRM) status (*Table S1*). In this meta-analysis, pathologically successful resection was defined as complete or near-complete TME, clear CRM (> 1 mm), and clear DRM (> 1 mm). Time points of interest were 3 years for LRR and DFS, and 5 years for OS.

### Statistical analysis

The analysis was performed according to the intention-to-treat principle where patients were analysed according to the surgical procedure to which they were randomized. Patients who received the alternative procedure either at commencement or after initiation of the allocated surgery were analysed according to the original allocation. Patients who were allocated the laparoscopic procedure but received a hybrid or open procedure were considered conversions. Patients with metastatic disease (M1) who were included in ALaCaRT (*n* = 23) were excluded from the DFS and OS analyses but included in the analysis of pathology endpoints. Patients who were randomized but refused surgery were excluded from the analysis of pathology endpoints. Patients with no residual tumour, in whom CRMs and DRMs were not measurable, were assessed according to completeness of TME grade only. Statistical significance was set at α = 0.05 (two-sided except for the non-inferiority test, which was one-sided). No adjustments were made for any multiple comparison.

DFS was measured from the date of randomization to the date of first recurrence or death. Patients who were alive and without recurrent disease or patients who were lost to follow up were censored at the date last known to be alive. Kaplan–Meier curves were used to estimate DFS for each trial and to provide estimates of the standard errors. The differences in 3-year DFS rates between trials were pooled to obtain a pooled 3-year DFS difference. The non-inferiority margin of laparoscopic surgery with respect to open surgery for DFS at least 3 years after surgery was an absolute difference of 5%, where non-inferiority would be declared if the lower bound of the one-sided 95% confidence interval (c.i.) for the absolute difference between surgical procedures exceeded this margin. The non-inferiority margin was prespecified and was based on 3-year DFS results from the COLOR II^8^ and COREAN^9^ trials and blinded to any long-term results from the ALaCaRT and ACOSOG Z6051 trials. For LRR and distant recurrence, a competing risk analysis using the method of Fine and Gray was performed^10^. Deaths that occurred in patients before local recurrence or evidence of distant disease were considered a competing risk. OS was analysed using the same methods as for DFS except that proportions at 5 years were determined.

Associations between treatment arm and pathological outcomes (composite successful resection and individual components) were analysed in logistic regression models adjusted for trial (where the trial was included as a covariate). Univariate analyses of association between clinicopathological factors and DFS/OS were obtained by proportional hazards regression stratified by trial and adjusted by treatment arm. Baseline characteristics and pathological outcomes (including the three individual components of pathologically successful surgery) were considered as candidates for a multivariable model for DFS and OS. Forward stepwise selection requiring *P* < 0.05 for entry and retention in the model was used to select the predictors in the final multivariable model. Treatment arm (laparoscopic or open surgery) was a required predictor in the multivariable model regardless of significance. There were no missing data for pathological outcomes. Data were missing for the following baseline characteristics: T category (2 patients, < 0.1%), N category (2, < 0.1%), tumour–anal verge distance (2, < 0.1%; not all the same two patients), and tumour size (80, 8.6%). Tumour size was excluded from consideration for inclusion in the multivariable models so that the final model could be fitted on maximum possible data. Univariate analysis of the association of tumour size with survival outcomes is reported. No missing data were imputed in the analysis.

## Results

Of the 475 patients in ALaCaRT, 473 were included in the meta-analysis and two were excluded (sigmoid tumour > 15 cm from the anal verge; no protocol surgery). Of these 473 patients, 235 (49.7%) underwent open surgery and 238 (50.3%) underwent laparoscopic surgery. The median follow-up was 60.5 (interquartile range (i.q.r.) 60.1–61.3) months. Of the 486 patients in Z6051, 462 patients were evaluable for the analysis of that trial’s primary endpoint (pathologically successful resection) and were included in the meta-analysis; 24 were excluded (15 withdrew consent, four with improper consent, two with metastasis, one who chose surveillance over surgery, one who refused open surgery, and one withdrawn by site (non-compliance)). Of these 462 patients, 222 (48.1%) underwent open surgery and 240 (51.9%) underwent laparoscopic surgery. The median follow-up was 50.4 months (i.q.r. 36.9–60.6). The combined data set therefore consisted of 935 patients: 457 (48.9%) who underwent open surgery and 478 (51.1%) who underwent laparoscopic surgery, with a median follow up of 60.2 (i.q.r. 49.9–61.1) months. Baseline characteristics according to randomization group for the pooled data set, as well as individual ALaCaRT and Z6051 trials, are presented in *Table 1* and *Table S2*, respectively.

**Table 1:** Baseline characteristics by treatment arm for the pooled data set of patients from the ALaCaRT and Z6051 trials

	Pooled laparoscopic	Pooled open
No. of patients	478	457
Age (years), mean(s.d.)	60.9(12.4)	60.4(12.8)
BMI (kg/m^2^), mean(s.d.)	26.7(4.3)	26.8(4.4)
Tumour distance from anal verge (cm), mean(s.d.)	7.1(3.4)	7.3(3.3)
Tumour size (cm), mean(s.d.)	3.7(2.0)	3.7(2.0)
Neoadjuvant radiotherapy	363 (75.9%)	341 (74.6%)
Abdominoperineal resection	73 (15.3%)	66 (14.4%)
Low anterior resection	405 (84.7%)	391 (85.6%)
Conversion to open	48 (10%)	
**Sex**		
Female	163 (34.1%)	159 (34.8%)
Male	315 (65.9%)	298 (65.2%)
**Tumour location**		
High rectum	85 (17.8%)	75 (16.4%)
Middle rectum	188 (39.3%)	193 (42.2%)
Low rectum	205 (42.9%)	189 (41.4%)
**Tumour stage**		
T0	0 (0.0%)	0 (0.0%)
T1	21 (4.4%)	11 (2.4%)
T2	80 (16.8%)	82 (18.0%)
T3	375 (78.6%)	363 (79.6%)
T4	1 (0.2%)	0 (0.0%)
**Nodal category**		
N0	206 (43.2%)	212 (46.4%)
N1	217 (45.5%)	198 (43.3%)
N2	53 (11.1%)	47 (10.3%)

Values are *n* (%) unless otherwise stated. s.d., standard deviation; BMI, body mass index.

### Pathology outcomes

**Figure 1:**
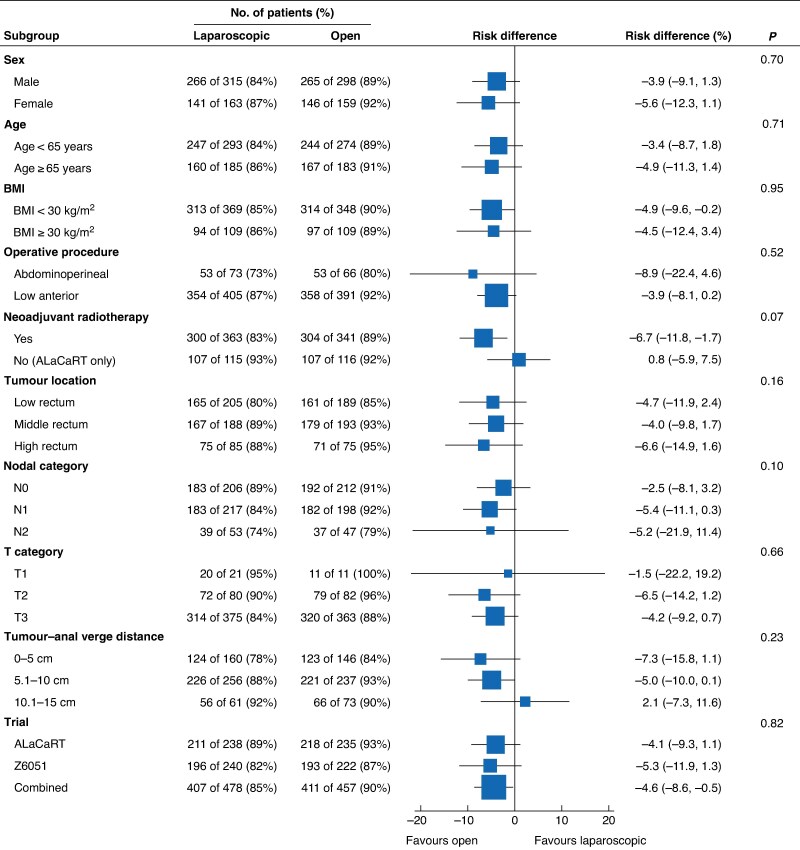
Rate of pathologically successful resection for all patients and major subgroups Values in parentheses are 95% confidence intervals unless otherwise stated. The *P* values are for tests of interactions except where there are more than two categories, in which case a test for linear trend was used. For neoadjuvant radiotherapy, the test of interaction uses ALaCaRT data only because all participants in the Z6051 trial received neoadjuvant radiotherapy. BMI, body mass index.

Individual pathology outcomes and the composite pathologically successful resection according to randomization group are presented in *Table 2*. The rate of pathologically successful resection was lower in the laparoscopic than open group (407 (85.1%) *versus* 411 (89.9%), respectively; odds ratio 0.645; 95% c.i. 0.434 to 0.960; *P* = 0.031). The pooled estimate of treatment effect demonstrated a 4.6% (95% c.i. −8.6 to −0.5) difference in pathologically successful resection favouring open surgery. The treatment effect of open *versus* laparoscopic surgery on pathologically successful resection by prespecified subgroups and individual trial is presented in *Fig. 1*. The treatment effects were similar for each trial without evidence of heterogeneity seen for any subgroup.

**Table 2:** Association between treatment arm and pathological outcomes

	Laparoscopic	Open	P*
Pathologically successful surgery	407 (85.1%)	411 (89.9%)	0.031
Circumferential resection margin (> 1 mm)	431 (90.2%)	426 (93.2%)	0.099
Distal resection margin (> 1 mm)	471 (98.5%)	452 (98.9%)	0.630
Complete/nearly complete TME	451 (94.4%)	444 (97.2%)	0.041
Complete TME	381 (79.7%)	397 (86.9%)	0.004

Values are *n* (%). TME, total mesorectal excision. *P values calulated using logistic regression models adjusted for trial, with included as a covariate.

### Disease recurrence and survival

**Figure 2:**
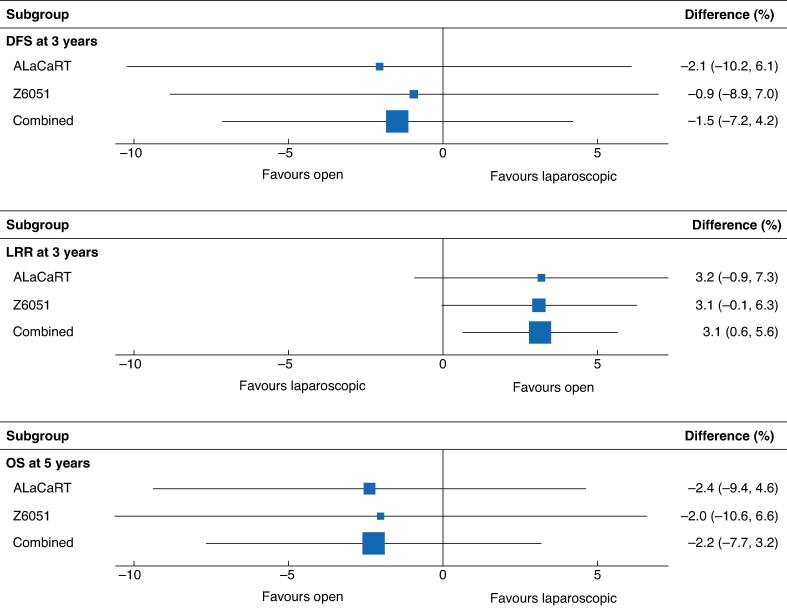
Forest plots showing differences in 3-year DFS, 3-year LRR and 5-year OS between treatment arms for individual studies and the pooled data set Values in parentheses are 95% confidence intervals. DFS, disease-free survival; LRR, locoregional recurrence; OS, overall survival.

The 3-year DFS was 75.2% (95% c.i. 71.1% to 79.2%) for the laparoscopic group and 76.5% (95% c.i. 72.5% to 80.6%) for the open group (pooled estimated difference −1.5%; 95% c.i. −7.2% to 4.2%; *Fig. 2*). Non-inferiority of laparoscopic surgery was not demonstrated given the non-inferiority margin of Δ = −5% was contained within the one-sided 95% c.i. for the pooled estimated difference (lower limit −6.3%). A sensitivity analysis excluding patients with high rectal tumours yielded similar results to the primary analysis, with a 3-year DFS difference of −2.0% (95% c.i. −8.2% to 4.3%) compared with −1.5% (95% c.i. −7.2% to 4.2%) in the full cohort.

The 5-year OS was 81.3% (95% c.i. 77.5% to 85.2%) for the laparoscopic group and 83.6 (95% c.i. 79.9% to 87.4%) for the open group (pooled estimate difference −2.2%; 95% c.i. −7.7% to 3.2%). After accounting for competing risks (distant recurrence and death), the 3-year LRR rate was higher in the laparoscopic group (5.4%; 95% c.i. 3.3% to 7.5%) than in the open group (2.0%; 95% c.i. 0.7% to 3.4%), with a pooled estimate of 3.1% difference (95% c.i. 0.6% to 5.6%).

### Association between pathology outcomes and survival

**Figure 3:**
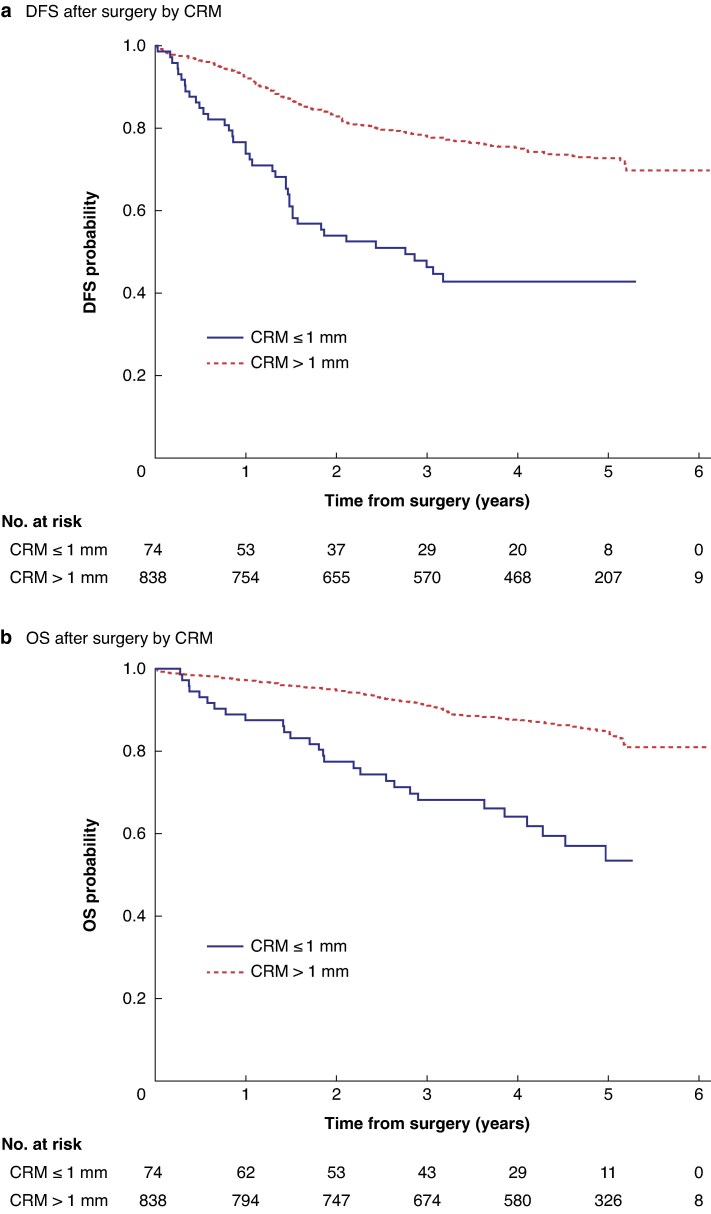
DFS and OS according to CRM status (pooled analyses, not adjusted for trial) **a** DFS (HR 0.32; 95% c.i. 0.23, 0.46; *P* < 0.001) and **b** OS (HR 0.28; 95% c.i. 0.18, 0.42; *P* < 0.001) after surgery according to CRM status. Kaplan–Meier plots were created using pooled data. HR estimates are stratified according to trial. DFS, disease-free survival; OS, overall survival; CRM, circumferential resection margin; HR, hazard ratio; c.i., confidence interval.

Both the composite pathologically successful resection and clear CRM were associated with improved DFS (*P* < 0.001 and *P* < 0.001, respectively) and OS (*P* < 0.001 and *P* < 0.001, respectively) in a univariate analysis adjusted for treatment arm and stratified by trial (*Fig. 3*). In a multivariable model, a clear CRM was the most significant predictor and the only pathology metric found to be a predictor of both DFS (hazard ratio (HR) 0.32; 95% c.i. 0.23 to 0.45; *P* < 0.0001) and OS (HR 0.26; 95% c.i. 0.17 to 0.39; *P* < 0.0001; *Tables S3* and *S4*).

## Discussion

This preplanned individual patient meta-analysis of the ALaCaRT and ACOSOG Z6051 randomized trials failed to demonstrate non-inferiority of laparoscopic *versus* open proctectomy for rectal adenocarcinoma in terms of 3-year DFS (the planned primary endpoint of the meta-analysis). Acknowledging that lack of power for this endpoint may partially explain this finding, which remains inconclusive, a poorer DFS is not excluded. Laparoscopic surgery led to a significantly lower rate of pathologically successful resection and a higher 3-year LRR rate compared with open proctectomy.

This prospective meta-analysis also demonstrated that the composite endpoint of pathologically successful resection (combining complete/near complete TME and clear CRM and DRM) used in both trials is associated with improved DFS and OS. Importantly, when the individual components of the composite endpoint were evaluated in a multivariable model with adjustment for trial, treatment arm, and other baseline risk factors, a clear CRM remained the most important independent predictor of DFS and OS. This demonstrates that, although the composite pathologically successful resection endpoint reflects surgical quality and is a potential surrogate marker of long-term oncological outcomes after proctectomy for rectal cancer, the primary driver of this effect may be CRM status. It is therefore reasonable to consider further evaluating CRM status alone, rather than as part of a composite pathological metric, as a potential surrogate for long-term oncological outcomes in future trials^6^.

It is possible that successful rectal cancer surgery involving proctectomy (in particular, achieving a clear CRM) reduces LRR but does not affect DFS or OS, similar to the well documented local oncological effect of radiotherapy for the treatment of rectal cancer^11,12^. However, the number of events in terms of recurrence and death were low, which led to wide confidence intervals for LRR and survival estimates. In view of the treatment effects favouring open surgery, and the demonstrated impact of laparoscopic surgery on pathological outcomes that strongly predict long-term outcomes, the possibility of poorer oncological outcomes after laparoscopic TME for rectal cancer in some patients requires further evaluation.

Two other contemporaneous randomized trials comparing laparoscopic and open rectal cancer surgery, which were not included in this meta-analysis, had somewhat different outcomes. The international COLOR II trial (1044 patients randomized 2 : 1 to laparoscopic : open surgery) reported no difference in pathological results, LRR, or DFS between laparoscopic and open groups, with DFS estimates favouring laparoscopic surgery (4% difference at 3 years) and 5% 3-year LRR in both groups^8^. However, patients with T3 tumours within 2 mm of the mesorectal fascia on preoperative imaging were excluded from COLOR II and therefore likely represent a population at inherently lower risk of margin involvement regardless of treatment arm. Despite this, a high rate of CRM involvement was reported in patients who underwent open surgery for low rectal tumours (22%) and likely influenced the outcomes. The COREAN trial^9^ (340 patients randomized 1 : 1) similarly reported non-inferiority of laparoscopic TME (3-year DFS difference 6.7%; 95% c.i. 15.8% to 2.4%; *P* < 0.001) favouring laparoscopic surgery. Importantly, the COREAN trial population had a lower mean body mass index and a significantly higher proportion of patients with early stage tumours with respect to both T category (T1/2) and nodal negative status compared with the ALaCaRT and Z6051 populations, which may explain, at least in part, the difference in results from the trials included in this IPD meta-analysis.

The findings of the present study are highly relevant when considered in the context of the recently reported long-term results of the REAL trial, which enrolled 1240 participants with middle and low rectal cancers (cT1–T3, N0–N1) and randomized them to robotic or laparoscopic proctectomy^13^. REAL is the first randomized trial to demonstrate the oncological superiority of robotic-assisted over conventional laparoscopic surgery for rectal cancer, with the primary endpoint of 3-year LRR significantly lower and the 3-year DFS significantly higher in the robotic group (1.6% *versus* 4.0% (HR 0.45, 95% c.i. 0.22 to 0.92, *P* = 0.03) and 87.2% *versus* 83.4% (HR 0.74; 95% c.i. 0.56 to 0.98; *P* = 0.04), respectively)^13^. Importantly, the rates of CRM clearance in the robotic and laparoscopic groups were 96.2% and 93.3%, respectively.

The 3-year LRR rate after laparoscopic surgery reported in REAL^13^ (4.0%) and in the present IPD meta-analysis (5.6%) are comparable, because they are comparing robotic surgery in the REAL trial (1.6%) and open surgery in the present study (2.5%). These improvements in LRR are likely attributable to the high quality of proctectomy that was achieved during robotic and open surgery in the respective trials, reflected by high rates of TME completeness and CRM clearance, overcoming the technical limitations of laparoscopy in certain situations. The REAL trial demonstrated improved rates of CRM and LRR in subgroups with high technical complexity, namely male patients, low tumours, and pathologically T3/T4a tumours^13,14^. Although differentially worse pathological outcomes of laparoscopic surgery for these subgroups could not be demonstrated in the present study, they were comparatively over-represented in the trials included in this IPD meta-analysis compared with REAL (which had a lower proportion of male participants, T3 tumours, and node-positive tumours, as well as a lower mean body mass index). It is expected that technological advances, including robotic-assisted TME, will overcome the limitations of laparoscopy in the pelvis and facilitate high-quality proctectomy involving TME dissection in more technically challenging patients, producing oncology outcomes comparable to open surgery.

Collectively, the findings of this study, in addition to the existing literature, emphasize the importance of precise surgical technique to achieve pathologically successful resection, and particularly a clear CRM, regardless of the surgical modality used (open or minimally invasive). Technical credentialing of surgeons was required in both ALaCaRT and ACOSOG Z6051 before the recruitment of patients and participation in trial procedures, which involved a minimum number of laparoscopic colectomies and proctectomies, and continuous audit of laparoscopic procedures during the trial. In ALaCaRT, although 30 previous laparoscopic TMEs was the requirement for surgeon eligibility, anecdotally five of the top six recruiting surgeons had experience with over 250 laparoscopic rectal dissections before trial commencement, and therefore represent a group of experienced laparoscopic surgeons. The relatively low number of conversions to open surgery and low frequencies of abdominoperineal resections and major complications (including anastomotic dehiscence) in both trials reflects the high quality of the rectal cancer surgery performed, particularly given the average body mass index of 27 kg/m^2^ and the high proportion of male patients. With the findings of lower rates of clear CRM and increased LRR in patients undergoing laparoscopic proctectomy in expert hands, concern about potentially poorer outcomes after surgery by less experienced surgeons is warranted. Furthermore, both trials were conducted before the popularity of total neoadjuvant therapy, which has recently been suggested to be the standard of care for locally advanced rectal cancer, but has also been associated with lower rates of complete TME and higher LRR after some total neoadjuvant therapy regimens, most likely due to the technical difficulty of surgery after a prolonged wait after radiotherapy^15^.

The limitations of this study include the fact that the ALaCaRT and Z6051 trials were similar, but not identical. For example, the combined pathology metric was defined differently in each trial with respect to both CRM and DRM. Fortunately, margins > 1 mm could be derived for individual patients in ALaCaRT from a measurement of the margin, facilitating a uniform combined pathology metric endpoint. Furthermore, both trials were originally powered to evaluate non-inferiority of the combined pathology metric, not long-term oncological outcomes including DFS, the primary endpoint of this meta-analysis, which may partially explain the inability to demonstrate non-inferiority. Differences between trial populations in terms of neoadjuvant treatment, tumour characteristics (including tumour height), and surgical complexity are unlikely to have confounded the overall treatment effect given that analyses were stratified by trial and based on intention-to-treat comparisons of randomized groups. However, subgroup analyses may have been underpowered to detect treatment effect heterogeneity according to these factors, including neoadjuvant radiotherapy. Details relating to the administration and completion of adjuvant chemotherapy were not available, and how these may have influenced the outcomes is unknown. However, if there were differences in chemotherapy administration based on pathological findings, this would likely favour a higher rate of adjuvant chemotherapy in the laparoscopic arm and hence lessen any potentially worse oncological outcome in the laparoscopic arm. The comparatively fewer laparoscopic proctectomies required for surgeon credentialing in Z6051 (20) may have meant that some surgeons were still on their learning curve and may have influenced outcomes in favour of open surgery. Potential short-term advantages of laparoscopy compared with open surgery, such as lower blood loss and shorter hospital stay and recovery time, were not evaluated in the present study. When interpreting the findings, it must also be considered that 3-year DFS does not always correlate with 5-year OS^16^.

In conclusion, this prospective IPD meta-analysis of the ALaCaRT and ACOSOG Z6051 trials demonstrates that laparoscopic surgery for rectal cancer led to a lower rate of pathologically successful resection and a higher rate of LRR at 3 years compared with open surgery. Although non-inferiority of laparoscopic surgery with respect to 3-year DFS was not demonstrated, this analysis was not specifically powered to detect differences in long-term oncological endpoint outcomes, and therefore definitive conclusions regarding DFS or OS cannot be drawn. Nevertheless, point estimates for both DFS and OS favoured open surgery, and the observed differences in pathological outcomes, particularly CRM status (which was strongly associated with survival), and the higher LRR raise the possibility of clinically meaningful oncological effects that require further evaluation. These findings emphasize the importance of precise oncological resection, particularly a clear CRM, regardless of surgical approach, and highlight the need for continued evaluation of surgical techniques and technologies that facilitate high-quality surgery for rectal cancer.

## Supplementary Material

zrag119_Supplementary_Data

## Data Availability

The data that support the findings of this study are stored securely at the University of Sydney and may be available from the corresponding author upon reasonable request. Access to the data will require approval from the ALaCaRT and Z6051 trial management executive committees and a University of Sydney data sharing agreement.
