# Supplementary material for: Laparoscopic versus open surgery for rectal cancer: individual patient data meta-analysis of the ALaCaRT and Z6051 randomized trials
Source: BJS Open. 2026 Aug 7;10(4):zrag119. doi: 10.1093/bjsopen/zrag119 (PMC13448722; doi:10.1093/bjsopen/zrag119)
Supplement: Supplementary file 1 [file zrag119_supplementary_data.docx]

**Title:**	Laparoscopic versus open surgery for rectal cancer: an individual patient data meta-analysis of the ALaCaRT and Z6051 randomized trials

**Authors:**

Kilian G. M. Brown PhD ^1,2,3^

Andrew R L Stevenson MBBS, ^4,5,6,7^

Michael J. Solomon DMedSc, DMed ^1,2,3^

James Murray MStat ^6^

Kate Wilson MPH ^2,3,6^

Val Gebski MStat ^6^

James Fleshman MD ^8^

Qian Shi PhD ^9^

Thomas E. Read MD ^10^

John Simes PhD ^6^

**Institutions:**

1. Department of Colorectal Surgery, Royal Prince Alfred Hospital, Sydney, Australia
2. Surgical Outcomes Research Centre (SOuRCe) and Institute of Academic Surgery, Royal Prince Alfred Hospital, Sydney, Australia
3. Faculty of Medicine and Health, Central Clinical School, The University of Sydney, Sydney, Australia
4. Royal Brisbane and Women’s Hospital, Brisbane, QLD, Australia
5. Faculty of Health, Medicine and Behavioural Sciences, University of Queensland, Brisbane, QLD, Australia
6. Faculty of Medicine and Health, National Health and Medical Research Council Clinical Trials Centre, University of Sydney, Camperdown, NSW, Australia
7. St Vincent’s Private Hospital Northside, Brisbane, QLD, Australia
8. Department of Surgery, Baylor University Medical Center, Dallas, Texas
9. Mayo Clinic, Rochester, MN, USA
10. University of Hawaii John A. Burns School of Medicine, Honolulu, Hawaii, USA

**Corresponding Author:** Dr Kilian G. M. Brown**,** Department of Colorectal Surgery, Royal Prince Alfred Hospital, Missenden Road, Sydney, NSW 2050, Australia. Phone: +61 2 9515 3200 Email : [kilian.brown@sydney.edu.au](mailto:kilian.brown@sydney.edu.au) ORCID ID: 0000-0003-0967-8515

| **Supplementary Table 1:** Characteristics of randomized trials included in the individual patient data meta-analysis.  **Supplementary Table 2.** Baseline characteristics by treatment arm for ALaCaRT and Z6051 | *page 4*  page 5 |
| --- | --- |
| **Supplementary Table 3.** Univariable and multivariable analysis of association between baseline/pathological characteristics and disease-free survival.  **Supplementary Table 4.** Univariable and multivariable analysis of association between baseline/pathological characteristics and overall survival | *page 6*  *page 7* |
|  |  |
|  |  |
|  |  |

**Supplementary Table 1:** Characteristics of randomized trials included in the individual patient data meta-analysis

|  | **ALaCaRT** | **ACOSOG Z6051** |
| --- | --- | --- |
| **Trial name** | The **A**ustralasian **LA**paroscopic **CA**ncer of the **R**ectum **T**rial | A Phase III Prospective Randomized Trial Comparing Laparoscopic-Assisted Resection vs Open Resection for Rectal Cancer |
| **Eligible cancer type** | Rectum | Rectum |
| **Intervention** | Laparoscopic-assisted resection | Laparoscopic-assisted resection |
| **Standard care** | Open resection | Open resection |
| **Trial Design** | Non-inferiority | Non-inferiority |
| **Primary Outcome** | Successful surgical resection defined by:  1. Circumferential margin ≥1mm  2. Distal margin ≥1mm  3. Completeness of TME | Adequate surgical resection defined by:  1. Circumferential margin >1mm  2. Negative distal margin  3. Completeness of TME |
| **Primary Endpoint Results** | Successful resection was achieved in 82% in the laparoscopic group and 89% in the open group. Did not support non-inferiority (risk difference of −7.0% [95% CI, −12.4% to 100%]; *P* = 0.38 for non-inferiority) | Successful resection occurred in 81.7% of laparoscopic group and 86.9% of open group. Did not support non-inferiority (risk difference, −5.3%; 1-sided 95%CI, −10.8% to ; *P* = 0.41 for non-inferiority). |
| **Sample size** | 470 | 480 |
| **Aspects of sample size**  **(Δ=Non-inferiority margin)** | Δ=0.08, one-sided α=0.05, 80% power  Based on 90% success rate in control group and accounting for up to 20% conversion rate | Δ=0.06, one-sided α=0.10, 80% power  Based on 90% success rate in control group |
| **Randomization** | 1:1 | 1:1 |
| **Stratification factors** | Site of tumor (high, middle, low), registering surgeon, planned operative procedure (low anterior resection, abdominoperineal resection), BMI (<30, ≥30), pre-operative radiotherapy, metastatic disease (M0 v M1) | Site of tumor (high, middle low), registering surgeon, planned operative procedure (low anterior resection, abdominoperineal resection) |
| **Countries of recruitment** | Australia and New Zealand | United States, Canada |
| **Eligible cancer stage** | T1-3, N0-2, M0-M1 | T1-3, N0-2, M0 |

**Supplementary Table 2.** Baseline characteristics by treatment arm for ALaCaRT and Z6051

| ***Baseline Characteristic*** | | ***ALaCaRT Laparoscopic*** | ***ALaCaRT***  ***Open*** | ***Z6051 Laparoscopic*** | ***Z6051***  ***Open*** |
| --- | --- | --- | --- | --- | --- |
| **No. (%)** | | 238 | 235 | 240 | 222 |
| **Age (years mean [SD])** | | 64.1 (12.5) | 63.6 (12.6) | 57.8 (11.5) | 57.0 (12.2) |
| **BMI (kg/m^2^ mean [SD])** | | 27.0 (4.5) | 26.8 (4.7) | 26.4 (4.0) | 26.8 (4.2) |
| **Tumor distance from**  **anal verge (cm mean [SD])** | | 8.0 (3.4) | 8.2 (3.3) | 6.1 (3.1) | 6.3 (2.9) |
| **Tumor size (cm mean**  **[SD])** | | 3.1 (1.6) | 3.1 (1.8) | 4.2 (2.2) | 4.3 (2.1) |
| **Neoadjuvant radiotherapy** | | 123 (51.7%) | 119 (50.6%) | 240 (100%) | 222 (100%) |
| **Abdominoperineal resection** | | 18 (7.6%) | 17 (7.2%) | 55 (22.9%) | 49 (22.1%) |
| **Low anterior resection** | | 220 (92.4%) | 218 (92.8%) | 185 (77.1%) | 173 (77.9%) |
| **Conversion to open** | | 21 (8.8%) | - | 27 (11.3%) | - |
| **Sex** | **Female** | 78 (32.8%) | 84 (35.7%) | 85 (35.4%) | 75 (33.8%) |
|  | **Male** | 160 (67.2%) | 151 (64.3%) | 155 (64.6%) | 147 (66.2%) |
| **Tumor Location** | **High rectum** | 53 (22.3%) | 50 (21.3%) | 32 (13.3%) | 25 (11.3%) |
|  | **Middle rectum** | 103 (43.3%) | 102 (43.4%) | 85 (35.4%) | 91 (41.0%) |
|  | **Low rectum** | 82 (34.5%) | 83 (35.3%) | 123 (51.3%) | 106 (47.7%) |
| **Tumor Stage** | **T0** | 0 (0.0%) | 0 (0.0%) | 0 (0.0%) | 0 (0.0%) |
|  | **T1** | 18 (7.6%) | 11 (4.7%) | 3 (1.3%) | 0 (0.0%) |
|  | **T2** | 68 (28.7%) | 68 (29.1%) | 12 (5.0%) | 14 (6.3%) |
|  | **T3** | 151 (63.7%) | 155 (66.2%) | 224 (93.3%) | 208 (93.7%) |
|  | **T4** | 0 (0.0%) | 0 (0.0%) | 1 (0.4%) | 0 (0.0%) |
| **Nodal status** | **N0** | 107 (45.1%) | 125 (53.2%) | 99 (41.3%) | 87 (39.2%) |
|  | **N1** | 92 (38.8%) | 80 (34.0%) | 125 (52.1%) | 118 (53.2%) |
|  | **N2** | 37 (15.6%) | 30 (12.8%) | 16 (6.7%) | 17 (7.7%) |

Abbreviations: BMI – body mass index, SD - standard deviation

**Supplementary Table 3.** Univariable and multivariable analysis of association between baseline/pathological characteristics and disease-free survival.

HR >1 indicates a shorter disease-free survival. Treatment arm (laparoscopic vs. open) was a required inclusion in the multivariable model but was not significant.

| **Level** | | **Univariate analyses** | | | **Multivariable model** | | |
| --- | --- | --- | --- | --- | --- | --- | --- |
|  |  | **HR** | **95% CI** | **p value** | **HR** | **95% CI** | **p value** |
| **Baseline Characteristics** | |  |  |  |  |  |  |
| Gender | Female | 1 |  |  |  |  |  |
|  | Male | 1.30 | (0.99, 1.70) | 0.06 |  |  |  |
| Nodal status | N0 | 1 |  | 0.06^*^ |  |  |  |
|  | N1 | 1.07 | (0.82, 1.40) |  |  |  |  |
|  | N2 | 1.58 | (1.07, 2.34) |  |  |  |  |
| Tumor location | Low rectum | 1 |  | 0.27^*^ |  |  |  |
|  | Middle rectum | 0.80 | (0.61, 1.06) |  |  |  |  |
|  | High rectum | 0.88 | (0.61, 1.25) |  |  |  |  |
| Tumor stage | T1 | 1 |  | 0.06^*^ |  |  |  |
|  | T2 | 1.27 | (0.57, 2.84) |  |  |  |  |
|  | T3 | 1.68 | (0.78, 3.61) |  |  |  |  |
| Operative Procedure | Abdominoperineal resection | 1 |  |  |  |  |  |
|  | Low anterior resection | 0.63 | (0.45, 0.87) | <.01 | 0.69 | (0.50, 0.96) | 0.03 |
| Neoadjuvant Radiotherapy (yes) | | 1.26 | (0.90, 1.77) | 0.17 |  |  |  |
| Tumor-anal verge distance (cm) | | 0.97 | (0.93, 1.01) | 0.08 |  |  |  |
| Tumor size (cm) | | 1.09 | (1.02, 1.16) | <.01 |  |  |  |
| Age (years) | | 1.02 | (1.01, 1.03) | <.01 | 1.02 | (1.01, 1.03) | 0.0002 |
| BMI (kg/m^2^) | | 0.99 | (0.96, 1.02) | 0.43 |  |  |  |
| **Pathological Outcomes** | |  |  |  |  |  |  |
| Pathologically adequate surgery | | 0.42 | (0.30, 0.57) | <.001 |  |  |  |
| Circumferential resection margin (>1mm) | | 0.32 | (0.23, 0.46) | <.001 | 0.32 | (0.23, 0.45) | <.0001 |
| Distal resection margin (>1mm) | | 0.58 | (0.24, 1.41) | 0.23 |  |  |  |
| Complete/Nearly complete TME | | 0.62 | (0.37, 1.05) | 0.08 |  |  |  |
| Complete TME | | 0.78 | (0.57, 1.08) | 0.13 |  |  |  |

TME - total mesorectal excision, HR - Hazard ratio, CI - confidence interval; * - Test of linear trend.

**Supplementary Table 4.** Univariable and multivariable analysis of association between baseline/pathological characteristics and overall survival.

HR >1 indicates a shorter overall survival. Treatment arm (laparoscopic vs. open) was a required inclusion in the multivariate model but was not significant.

| **Level** | | **Univariate analyses** | | | **Multivariate model** | | |
| --- | --- | --- | --- | --- | --- | --- | --- |
|  |  | **HR** | **95% CI** | **p value** | **HR** | **95% CI** | **p value** |
| **Baseline Characteristics** | |  |  |  |  |  |  |
| Gender | Female | 1 |  |  |  |  |  |
|  | Male | 1.17 | (0.83, 1.66) | 0.36 |  |  |  |
| Nodal status | N0 | 1 |  | 0.19^*^ |  |  |  |
|  | N1 | 1.09 | (0.77, 1.55) |  |  |  |  |
|  | N2 | 1.47 | (0.88, 2.46) |  |  |  |  |
| Tumor location | Low rectum | 1 |  | 0.07^*^ |  |  |  |
|  | Middle rectum | 0.72 | (0.50, 1.03) |  |  |  |  |
|  | High rectum | 0.71 | (0.44, 1.15) |  |  |  |  |
| Tumor stage | T1 | 1 |  | 0.54^*^ |  |  |  |
|  | T2 | 1.12 | (0.43, 2.92) |  |  |  |  |
|  | T3 | 1.24 | (0.50, 3.09) |  |  |  |  |
| Operative Procedure | Abdominoperineal resection | 1 |  |  |  |  |  |
|  | Low anterior resection | 0.46 | (0.31, 0.68) | <.001 | 0.55 | (0.37, 0.82) | 0.003 |
| Neoadjuvant Radiotherapy (yes) | | 1.13 | (0.73, 1.75) | 0.59 |  |  |  |
| Tumor-anal verge distance (cm) | | 0.93 | (0.89, 0.98) | 0.007 |  |  |  |
| Tumor size (cm) | | 1.13 | (1.05, 1.22) | 0.002 |  |  |  |
| Age (years) | | 1.06 | (1.04, 1.07) | <.001 | 1.06 | (1.04, 1.07) | <.0001 |
| BMI (kg/m^2^) | | 0.98 | (0.94, 1.02) | 0.30 |  |  |  |
| **Pathological Outcomes** | |  |  |  |  |  |  |
| Pathologically adequate surgery | | 0.40 | (0.27, 0.59) | <.001 |  |  |  |
| Circumferential resection margin (>1mm) | | 0.28 | (0.18, 0.42) | <.001 | 0.26 | (0.17, 0.39) | <.0001 |
| Distal resection margin (>1mm) | | 0.88 | (0.22, 3.54) | 0.85 |  |  |  |
| Complete/Nearly complete TME | | 1.01 | (0.44, 2.30) | 0.98 |  |  |  |
| Complete TME | | 0. 83 | (0.55, 1.26) | 0.38 |  |  |  |

TME - total mesorectal excision, HR - Hazard ratio, CI - confidence interval; * Test of linear trend.
